# Supplementary material for: Grapevine scion gene expression is driven by rootstock and environment interaction
Source: BMC Plant Biol. 2023 Apr 22;23:211. doi: 10.1186/s12870-023-04223-w (PMC10122299; doi:10.1186/s12870-023-04223-w)
Supplement: Supplementary file 1 — Additional file 1: Supplemental Figure 1. Experimental Design. A) Vineyard layout. The vineyard contains the grapevine cultivar Chambourcin grown ungrafted and grafted to three commercial rootstocks: 1103P, 3309C, and SO4. Each row of the vineyard contains all rootstock/scion combinations and is treated with one of three irrigation regimes: full (100% replacement of evapotranspiration), partial (50% replacement of evapotranspiration), or none (no replacement of evapotranspiration). B) Each cell of the vineyard features 4 replicated vines. Samples (leaf and reproductive) were collected from the middle 2 vines in each cell. This figure is partially adapted from [10], which is provided under the Creative Commons license (CC BY 4.0). [file 12870_2023_4223_MOESM1_ESM.pdf]

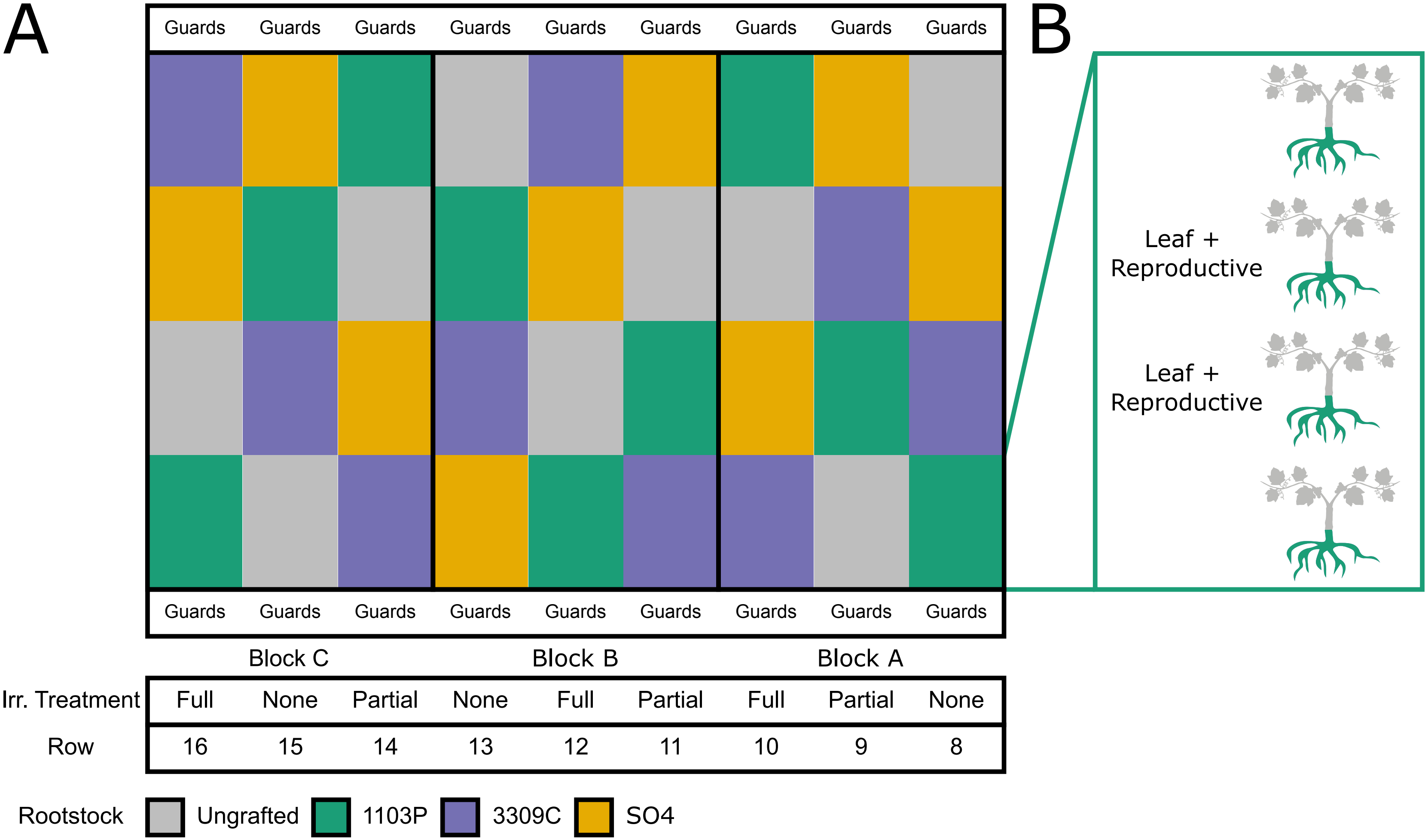

Supplemental Figure 1: Experimental Design. A) Vineyard layout. The vineyard contains the grapevine cultivar Chambourcin grown ungrafted and grafted to three commercial rootstocks: 1103P, 3309C, and SO4. Each row of the vineyard contains all rootstock/scion combinations and is treated with one of three irrigation regimes: full (100% replacement of evapotranspiration), partial (50% replacement of evapotranspiration), or none (no replacement of evapotranspiration). B) Each cell of the vineyard features 4 replicated vines. Samples (leaf and reproductive) were collected from the middle 2 vines in each cell. This figure is partially adapted from [10], which is provided under the Creative Commons license (CC BY 4.0).
