# Supplementary material for: Grapevine scion gene expression is driven by rootstock and environment interaction
Source: BMC Plant Biol. 2023 Apr 22;23:211. doi: 10.1186/s12870-023-04223-w (PMC10122299; doi:10.1186/s12870-023-04223-w)
Supplement: Supplementary file 3 — Additional file 3: Supplemental Figure 2. Example correlations between gene expression PCs and environmental PCs which differed by tissue. ePC1 and ePC2 are shown against the gPCs for which they explained large proportions of variation. [file 12870_2023_4223_MOESM3_ESM.pdf]

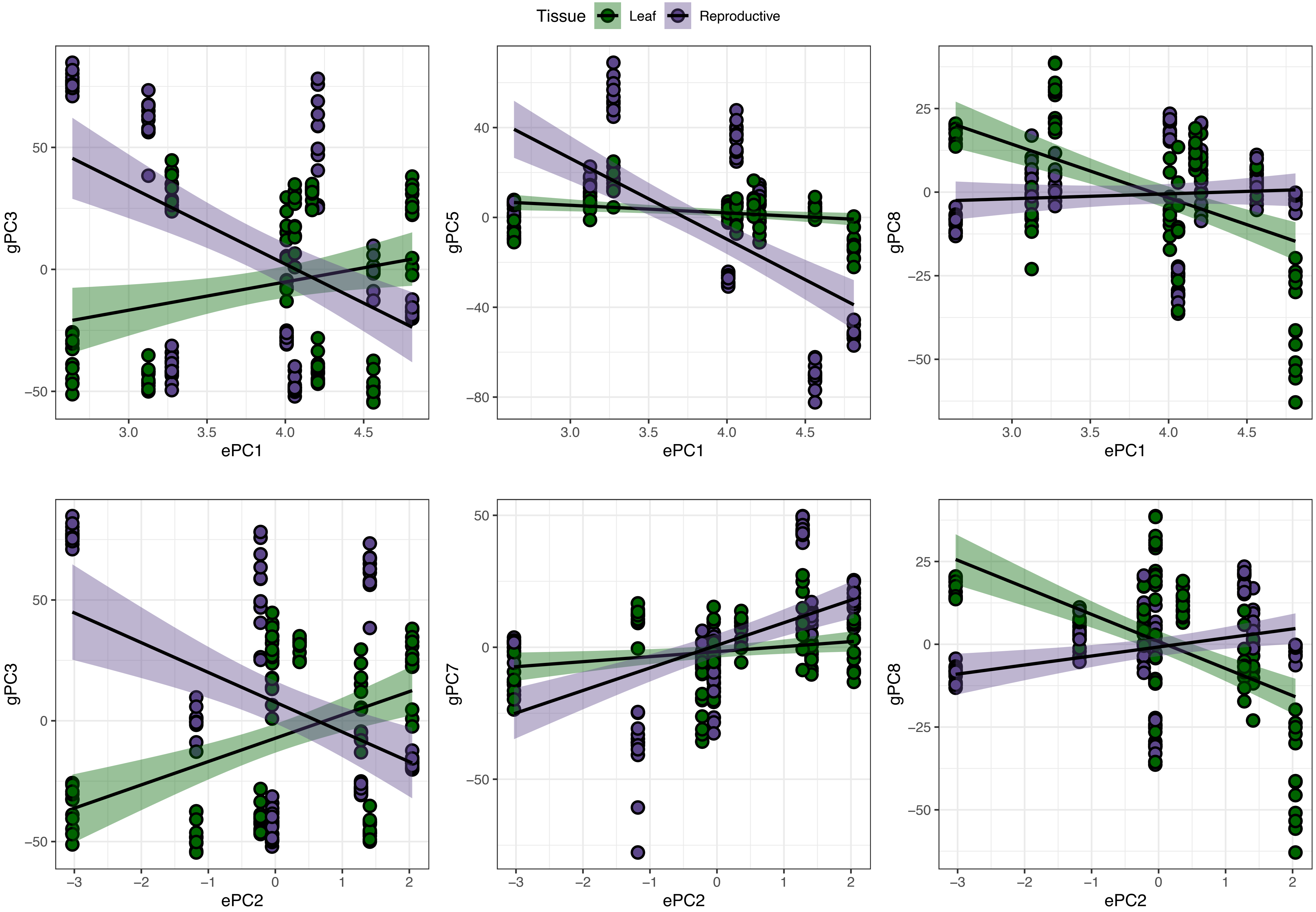

Supplemental Figure 2: Example correlations between gene expression PCs and environmental PCs which differed by tissue. ePC1 and ePC2 are shown against the gPCs for which they explained large proportions of variation.
