# Supplementary material for: Grapevine scion gene expression is driven by rootstock and environment interaction
Source: BMC Plant Biol. 2023 Apr 22;23:211. doi: 10.1186/s12870-023-04223-w (PMC10122299; doi:10.1186/s12870-023-04223-w)
Supplement: Supplementary file 4 — Additional file 4: Supplemental Figure 3. Survey of housekeeping genes. Two classes of housekeeping genes (Actin (IPR004000) and Ubiquitin (IPR000626)) were plotted against the major factors in the experiment's design (tissue, year, phenological stage, and rootstock genotype). Factor names are abbreviated to the first character of their name (Leaf: L, Reproductive: R, Anthesis: A, Veraison: V, Harvest: H, Ungrafted: U, 1103P: 1, 3309C: 3, SO4: S). [file 12870_2023_4223_MOESM4_ESM.pdf]

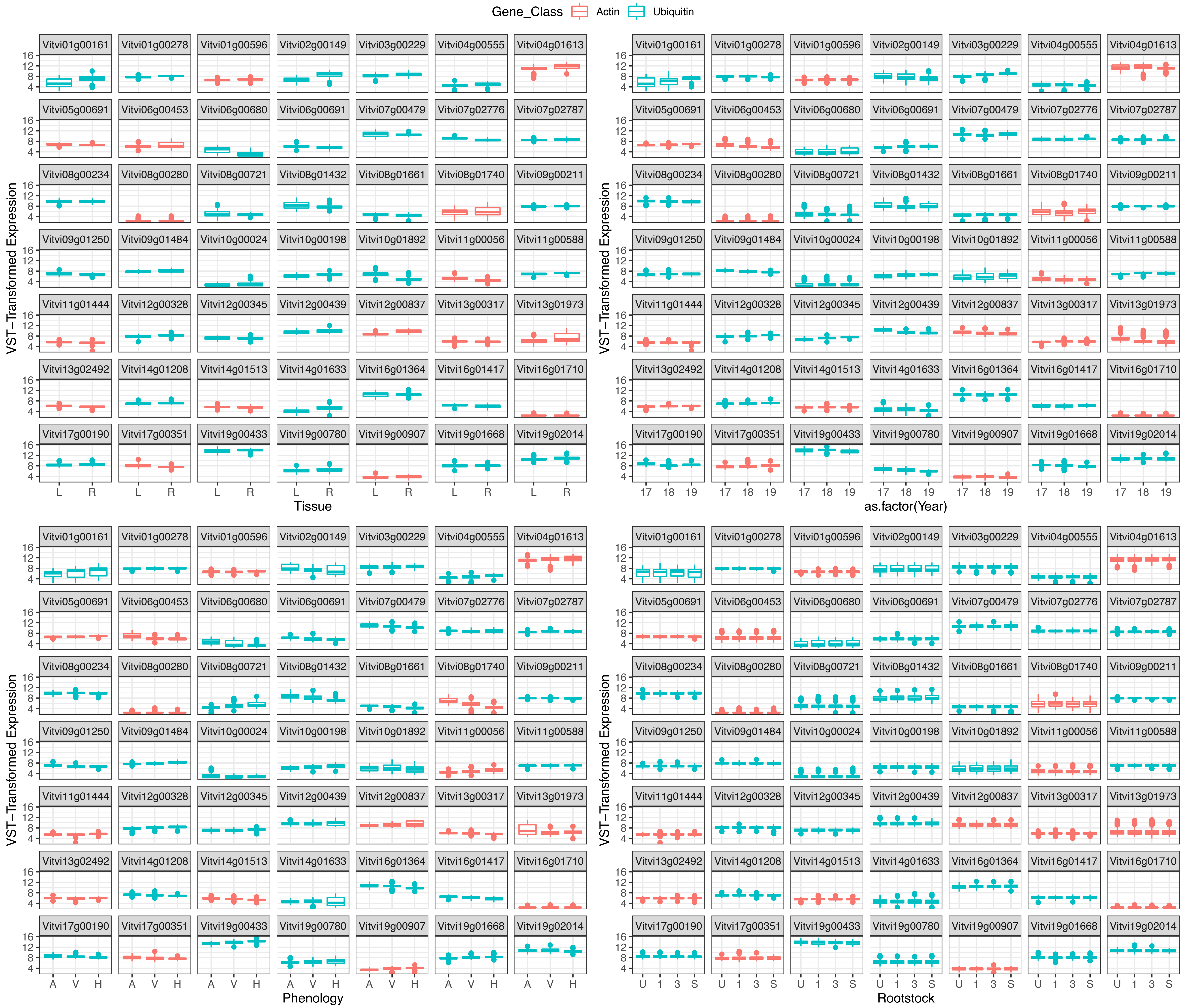

Supplemental Figure 3: Survey of housekeeping genes. Two classes of housekeeping genes (Actin (IPR004000) and Ubiquitin (IPR000626)) were plotted against the major factors in the experiment's design (tissue, year, phenological stage, and rootstock genotype). Factor names are abbreviated to the first character of their name (Leaf: L, Reproductive: R, Anthesis: A, Veraison: V, Harvest: H, Ungrafted: U, 1103P: 1, 3309C: 3, SO4: S).
